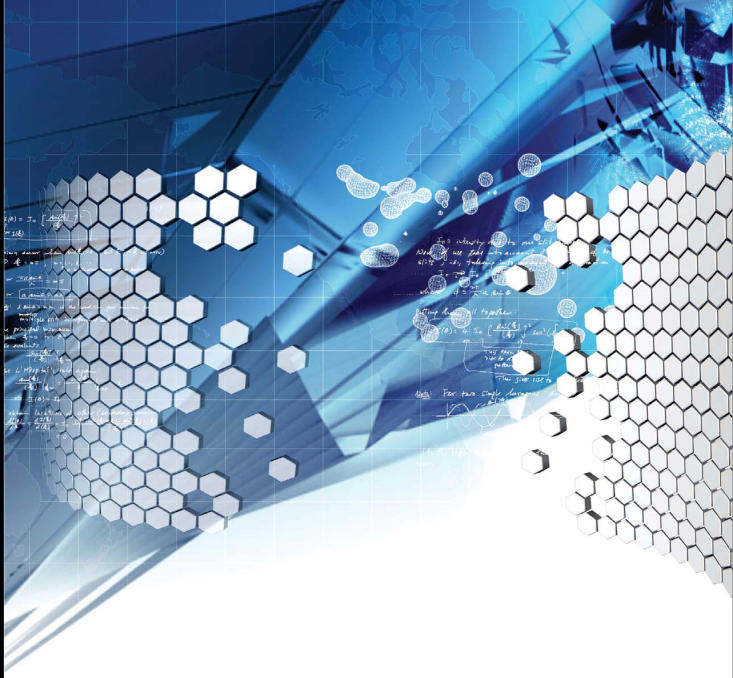# From Point B To Point A: Applying Toxicogenomics to Biological Inference

**DOI:** 10.1289/ehp.113-a388

**Published:** 2005-06

**Authors:** Kris Freeman

The data points in an integrated toxicogenomics experiment with microarray, proteomics, and metabolomics data are virtually innumerable. With thousands—or tens of thousands—of data points for each sample in each type of analysis, the complexity and the sheer amount of data multiply fast. As teams of statisticians, bioinformaticists, and biologists work to interpret this complexity, they must also ensure that each data point is valid and can be integrated with other data from the same or different types of experiments. Underlying this detailed exercise are two expansive goals. One is to identify markers of toxic exposures or disease. The other is to understand the biological processes underlying disease. The latter is called biological inference—the highly iterative process of inferring cause-and-effect relationships from toxicogenomics data, using computation efforts linked to mathematics.

Efforts to identify markers of exposure are concerned primarily with discerning patterns in output from microarray, proteomics, and metabolomics technology. These patterns can be characterized as molecular fingerprints and can be extremely useful in diagnosing levels of exposure, even though researchers may not understand exactly why particular patterns appear. In contrast, biological inference is concerned with an understanding of how patterns in genomics data actually translate into the details of gene transcription, protein creation, and metabolism.

By studying associations among the expression of genes, proteins, and metabolites, researchers try to identify genes of influence, many of which act as hubs of metabolic networks, affecting many other genes. Transient hubs, those that act briefly as a cell changes state, can be especially difficult to find and analyze. A goal of special importance to toxicogenomics is to distinguish endogenous pathways—those involved in the cell’s normal chores of metabolism and reproduction—from exogenous pathways triggered by exposures to drugs or toxicants. The ultimate goal is to follow a pathway from the expression of genes through the creation and modification of proteins and metabolites, as well as all the associated gene–gene, protein–protein, and metabolite–metabolite interactions in between.

## Anchor Management

Inferring biological pathways requires research teams to mine and interpret vast quantities of genomics data. Interpretation strategies include grouping or clustering data to find patterns as well as use of statistical methods to filter data on genes with the strongest signals or those expressed in concert. One key technique in biological inference is phenotypic anchoring, using known biological information to interpret signals, or uncharacterized data, from genomics experiments. These signals indicate the presence of molecules (such as mRNA or proteins in a given range of molecular weights) and can take various forms, depending on the type of technology used. For example, in microarray experiments signals take the form of fluorescence generated by the bonding of strands of mRNA to the microarray slide. In some studies, these genomics data are compared to data from traditional toxicology tests performed on the same samples. In others, the team integrates what is already known or suspected about biological pathways based on past studies.

Examples of research using phenotypic anchoring can be found in acetaminophen studies sponsored by the NIEHS National Center for Toxicogenomics (NCT). In this research, microarray data were compared to those from traditional toxicology tests, which both aided in interpretation of the microarray data and led to the development of new knowledge and understanding about the toxicity of this commonly used drug.

For example, in one study published in the July 2004 issue of *Toxicological Sciences*, data from microarrays and traditional toxicity tests confirmed previous results from other labs showing that toxic doses of acetaminophen deplete adenosine triphosphate (ATP; molecules that store cellular energy) and damage mitochondria, the organelles that produce ATP. In addition, microarray data revealed other exposure effects that traditional tests had missed. For example, liver cells begin to express genes consistent with cellular energy loss at doses too low to cause the kind of cell damage that can be detected by histopathology and other traditional methods.

The microarray data also provided information on a possible new signature of acetaminophen toxicity involving the metal-lothionein gene and several others, which may be involved in the liver’s antioxidant defense system. “We didn’t previously know that those genes were involved in acetaminophen toxicity, but it fits into the biological story of cell defense mechanisms,” says Alexandra Heinloth, a research scientist with the NCT and lead author of the 2004 paper.

A different type of phenotypic anchoring was used in a study of pathways linked to inflammation. Microarray experiments with mouse strains that exhibited both high and low levels of response to inhaled lipopolysaccharide compounds (which trigger immune responses) identified about 500 genes that were responsive in at least one of the strains. Researchers from Duke University, The Institute for Genomic Research, and George Washington University, including John Quackenbush, now a professor in biostatistics and computational biology at the Dana-Farber Cancer Institute and the Harvard School of Public Health, used two independent methods to filter the results of these microarray experiments, to prioritize genes for future study.

In the first method, the team identified 30 genes whose expression levels best distinguished the low- and high-responding mice. In the second method, they used quantitative trait locus (QTL) analysis to find regions genetically linked to the strength of the lipopolysaccharide-induced response. When the researchers compared their 500-gene list to the QTL regions, they found a set of 28 that were both differentially expressed and genetically linked to the observed phenotypes. There was no overlap among the genes identified by these two methods.

What people can find across many arrays are patterns suggesting coregulation. If you look across hundreds of arrays and find that expression of two genes [moves] up and down together, that’s highly suggestive of interactive behavior. It’s not so much biological modeling as it is finding associations that are suggestive of biological interactions.

–Terry Speed

University of California, Berkeley

In a report of the study published in the June 2004 issue of *Genomics*, the researchers acknowledge that they may miss genes with important roles with these filtering methods. They argue, however, that their approaches provided an objective way to obtain a small number of high-priority genes for future functional studies.

## Beyond Microarrays

Although researchers aim to eventually link pathways from expression to metabolism, genomics research thus far has focused on microarrays because this technology is more standardized and far more widely available than methods for analyzing proteins (primarily mass spectrometry) and metabolites (mass spectrometry and nuclear magnetic resonance). Although array-like assays for proteins have been developed, some using antibodies as tags, these technologies are still relatively exploratory and limited in scope, says Terry Speed, a professor in the Department of Statistics at the University of California, Berkeley.

But microarray data have serious limitations when it comes to biological inference. Although they can show associations, such data can rarely indicate cause and effect. “What people can find across many arrays are patterns suggesting coregulation,” says Speed. “If you look across hundreds of arrays and find that expression of two genes [moves] up and down together, that’s highly suggestive of interactive behavior. It’s not so much biological modeling as it is finding associations that are suggestive of biological interactions.” One of the greatest limitations of microarray data reflects the underlying biology: the expression of mRNAs doesn’t always translate into proteins because silencing RNAs and other mechanisms can block the translation process.

Sample sizes in most of the current systems biology experiments are not adequate to infer the kinds of complex networks that are the goal of such studies.

– Gary Churchill

Jackson Laboratory

One way to bridge the gap between gene expression and protein creation is to assess the proteins in a cell through proteomics analysis. Another is to gain a better understanding of the “transcriptome” (also called the “RNAome”)—the expression of all regulatory elements operating to regulate the expression, stability, and translation of transcripts (strands of RNA) in the cell. “To make genotype–phenotype correlations, you need to have a complete catalogue of transcripts that are expressed at each locus where such genotype–phenotype correlations are to be made,” says Thomas Gingeras, vice president of biological science at Affymetrix.

Gingeras and other researchers at Affymetrix and the National Cancer Institute have studied the RNAome with microarrays containing probes for the entire nonrepetitive sequences, not just the coding regions, of 10 human chromosomes. Data from these arrays have demonstrated that RNA activity is extraordinarily varied and complex. Although researchers have been able to identify the roles of many sequences, such as ribosomal and protein-coding RNAs, they have had to classify a substantial number of the newly discovered transcripts as TUFs (transcripts of unknown function).

Sequences for TUFs are equally complicated. “Curiously enough, many of these transcripts are sitting in the middle of genes, overlapped on both the sense and antisense strands of coding sequences,” says Gingeras. The sense, or template, strand of DNA is the one that is copied or transcripted. The authors speculate that the RNAs correlating to antisense strands may be cRNA copies, created in somewhat the same way as the cDNA copies of RNA used in microarrays.

Another challenge in all types of genomics experiments is detecting signals from molecules expressed at low levels. Quantities of mRNAs and proteins in a sample can vary by a ratio of 1 million to 1. Some of these low-expression molecules could be critical triggers to biological cascades, but may be lost in the signal-to-noise ratio.

Proteomics technologies are making progress in detecting low-expression molecules through more sophisticated sorting technologies such as SELDI-TOF (a method that selects only a subset of proteins from a given sample for analysis). To detect low-expression transcripts and quantify the number of mRNAs in a given sample, researchers studying gene expression turn to methods such as RT-PCR (which can involve the use of controls and fluorescent markers to quantify the amount of a molecule produced during polymerase chain reaction) and SAGE (which involves marking each transcript with a unique tag and then linking and sequencing the combined transcripts to count the number of times each tag occurs).

## Analysis Issues

As great as the challenges are in developing technology to detect and identify transcripts, proteins, and metabolites, the difficulties in analyzing the resulting data may be even greater. As research teams plan their experiments, they must chose from a bewildering and ever-changing assortment of statistical methods for data analysis. Speed says no one protocol will work for all experiments: “Usually there will be one method that will be preferred and often several that will be acceptable. All of them have their strengths and weaknesses.”

Part of the difficulty relates to the current nature of genomics experiments. Whereas traditional statistics methods are based on the assumption that a study will have far more samples than data points per sample, genomics experiments usually involve the inverse situation: a few dozen samples, with tens of thousands of data points per sample. New statistical methods are being developed to deal with the peculiarities of genomics data. Simultaneously, other teams are working to ensure that the data to be analyzed are valid by addressing issues such as differences in experimental technologies and laboratory procedures, and revisiting the effects of sample size.

Recent studies have shown that increased standardization of microarray platforms has greatly reduced the influence of platform type on results. In a study published in the May 2005 issue of *Nature Methods*, Quackenbush and colleagues found that differences in microarray platforms (oligonucleotide versus spotted cDNA) did indeed affect results. However, these differences were eclipsed by a very high correlation between the platforms in expression changes caused by varying exposures to angiotensin II, a potent peptide that causes blood vessels to constrict. “The question we asked was, does the biology or the platform dominate?” says Quackenbush. “For more than ninety percent of genes for which we could make a reasonable comparison, we found that biology dominated platform.”

The bad news is that variations in protocols (for RNA labeling, hybridization, and microarray processing), statistical methods for data acquisition and normalization, and other “lab effects” can still significantly impact microarray data. This was the finding of two other studies also published in the May 2005 issue of *Nature Methods*, one led by Rafael Irizarry, an associate professor in the Department of Biostatistics at The Johns Hopkins University, and another by researchers with the NIEHS Toxicogenomics Research Consortium (TRC). Both studies compared the results of microarray analysis of identical samples performed at multiple laboratories, and both found that, with care, results can be comparable across labs. However, “you have to pay close attention to how you do things. You have to standardize your protocols from lab to lab,” says Katherine Kerr, a coauthor of the TRC study and the director of the Bio-informatics and Biostatistics Facility Core at the University of Washington–NIEHS Center for Ecogenetics and Environmental Health.

The design for most microarray experiments calls for three to five samples per treatment condition. However, some statisticians say that sample size must be increased to generate the statistical power necessary to infer biological pathways. “Sample sizes in most of the current systems biology experiments are not adequate to infer the kinds of complex networks that are the goal of such studies,” says Gary Churchill, a staff scientist at the nonprofit Jackson Laboratory in Bar Harbor, Maine.

Churchill is a cofounder of the Collaborative Cross, a project to develop a panel of 1,000 new and genetically diverse mouse strains. The mice are descendants of just eight parent strains, minimizing the need for genotyping, and are being bred for maximum genetic variation, allowing for a plethora of diverse yet controlled strains. Not all studies will require 1,000 mice, Churchill says, although some will. The resource is being generated to cover a wide range of needs.

Statistics isn’t about the formulas, how to crunch numbers. It’s about the concepts. It’s about how to quantify uncertainty, about how to take data and turn it into knowledge.

– Gary Churchill

Jackson Laboratory

## Interpretation Is Key

Before teams can begin analyzing data, they must be confident that they are interpreting the raw signals accurately. For microarray data, this involves summarizing the fluorescence data for each spot—which are generated by the individual mRNAs linked to the probes—into a single value for each gene. “The hardest challenge is removing the component of intensity that is due to background noise,” says Irizarry. Some background noise—extraneous signals that can be confused with the signals being observed—can be caused by mismatches in the attachment of mRNA to the chip.

Another potential issue, says Irizarry, is that some of the 25–base pair probes used on oligonucleotide chips can be “stickier” than others—that is, more likely to attract mRNA. “If one gene is represented by a sequence that’s sticky, it will collect more than a probe that is less sticky,” he says. As a result, results from such a probe may reflect the chemistry of cDNA–mRNA bonding more than the biology of the sample.

Once values have been established for each gene in an array, the signals need to be normalized across the array. Equalizing fluorescence signals in two-color arrays is the most basic type of normalization. If equal amounts of two samples were hybridized to an array, then the total fluorescent signal from each sample should also be equivalent. If one is uniformly higher, a statistician can adjust the fluorescence values to better represent the relationship between the samples. Several more-involved processes may also be used to normalize data.

According to the TRC study, normalization procedures seem to increase the accuracy of microarray data. But there still remain lots of unanswered questions about normalization formulas, as well as the algorithms used to analyze genomics data, says Kerr. “The data look better when we’re done with [normalization],” she says. “But we don’t know if we’ve really made a correction.”

After normalization, researchers can take a basic count of changes in gene expression. Churchill calls this “making lists.” He explains: “You measure microarray data from normal tissue and from diseased tissue and . . . generate lists of genes that are up or down. The problem now is how we take those lists and turn them into biological sense.”

## Turning Data into Sense

The next step for many teams is to shorten the list. They may focus on genes with the strongest or most closely correlated changes in expression. Often this process is informed by preexisting information about gene function. But care must be taken. Setting filters that are too tight can cause an analysis to ignore genes of importance, while setting parameters too broadly can cause false positives.

That is why different groups can have difficulty replicating results on microarray experiments, says Greg Carr, a research fellow working in product safety at Procter and Gamble. If the statistical power—or probability of detecting genes of significance—is set relatively low, say 10%, one lab may pick up on some of the low-power genes and a second lab may pick up on others, but no one lab is likely to detect them all, he says.

We need to simplify to understand complexity, to start off with the exploration and understanding of simple systems. If you try to look at the complexity first off, you’ll never really unravel it.

– Kenneth Ramos

University of Louisville

Another approach sometimes used with or instead of data filtering is to cluster, or group, data according to similarities in expression patterns. Methods include hierarchical or “Eisen” clustering, which produces a tree-like structure; κ-means clustering, which produces line graphs; and principal components analysis, which produces a three-dimensional array that can be rotated. Clustering methods are usually exploratory and, Speed says, don’t provide an answer to a well-defined question; they can only show associations among genes. However, they can provide effective ways to organize data. Scientists then have to infer cause and effect.

Various types of modeling algorithms can aid in this inference process. One of the simplest models, Boolean networks, can capture multivariate gene relationships that can be inferred from measurement data. Ilya Shmulevich, an associate professor at the Institute for Systems Biology in Seattle, has worked to increase the flexibility of Boolean network modeling through the development of probabilistic Boolean networks. These networks allow for multiple functional possibilities for each gene, mimicking underlying biological and measurement uncertainty, says Shmulevich. He and his colleagues have applied probabilistic Boolean network analysis to gene expression data from studies of melanomas and gliomas.

When using most modeling methods, “you have to put in a rather limited set of genes and then learn something about that set,” says Speed. The art of assigning genes to modeling programs and deciding how to filter the results of other genomics data draws heavily on preexisting knowledge about the systems in general. Researchers comb through the scientific literature or databases of genetics, proteomics, and metabolomics data. However, database mining can only take researchers so far. Only 60–65% of human genes have been adequately annotated as to function, says Raymond Tennant, director of the NCT. This makes it difficult to infer the function of genes that haven’t yet been annotated.

The Chemical Effects in Biological Systems Knowledgebase, set to become publicly available in late 2005, will provide access to microarray data for about 140 reference compounds, and comprehensive data sets on about 10 hepatotoxicants, including acetaminophen. “The database will also include reference information on the biological effects of chemicals and other agents, and pathways related to their mechanism of action,” says Michael Waters, NCT assistant director for database development.

## Speaking the Language of Inference

Beyond having data in hand, biological inference requires a wide range of skills and expertise. “There’s a need for transdisciplinary efforts—notice I said transdisciplinary rather than interdisciplinary,” says Kenneth Ramos, chair of the Department of Biochemistry and Molecular Biology at the University of Louisville and *EHP*’s toxicogenomics editor. “We need people who speak more than one language, a new type of scientist. We’ve become so specialized that it is difficult to cross disciplines with fluidity. I think that research in biological inference will demand that ability. Classically trained biologists will need to reengage their appreciation and understanding of mathematics in order to begin to tackle some of these questions.”

Biologists may also need to have a better understanding of, and possibly training in, statistics. “Taking Stats 101 isn’t necessarily going to imbue the concepts you need,” says Churchill. “Statistics isn’t about the formulas, how to crunch numbers. It’s about the concepts. It’s about how to quantify uncertainty, about how to take data and turn it into knowledge.”

“It would be beautiful if you had that [statistical] training when you start in the field,” says Heinloth. “But if you include enough statisticians and bioinformaticists on your team as equal members, you can have that covered.” In Heinloth’s research group, statisticians are involved in experiments from the beginning. “There’s hardly any decision in a study that’s made by only one person,” she says. However, she notes that this collaborative process is not “science by committee.” As experiments are designed and implemented, team members weigh in only in their areas of expertise.

As researchers delve into this enormous quantity of data, they are confronted with the limits of human cognitive ability. It is not possible for a single individual to fully comprehend the astonishing complexity of metabolism in even a single cell type. So as researchers develop new technologies and new statistical tools for genomics research, and for inferring the ramifications of the data they uncover, they also need to find new ways to approach the limits of their own understanding.

“We need to simplify to understand complexity, to start off with the exploration and understanding of simple systems. If you try to look at the complexity first off, you’ll never really unravel it,” says Ramos. There’s a lot of potential for error in starting with simple systems, he adds. But it’s a way to start, to gradually build up to more complex models.

“As we accumulate more data, we’re understanding how limited our understanding is and how much more there is to discover,” says Churchill. “That can be discouraging from a diagnostic sense, but it’s wonderful to have a new universe opening up in front of you.”

## Figures and Tables

**Figure:**
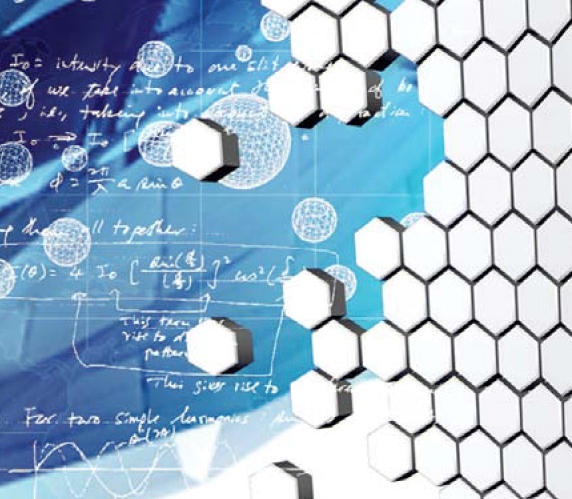


**Figure:**
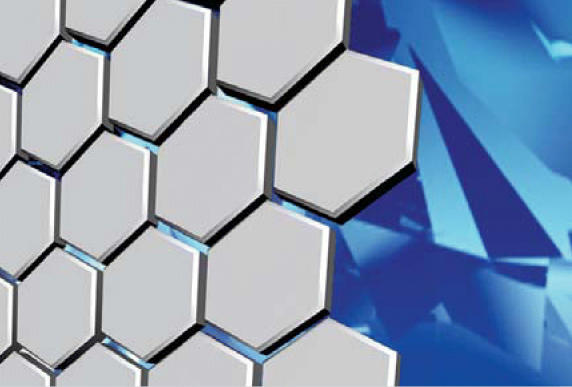


**Figure:**
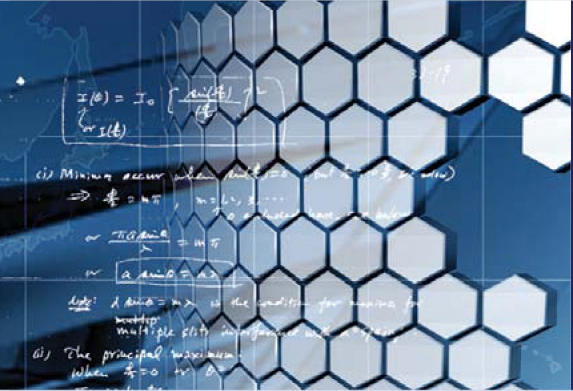


**Figure:**
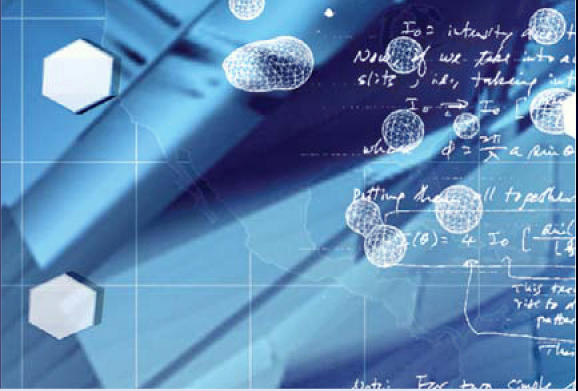


**Figure:**